# The Institutional Worker

**Published:** 1912-06-15

**Authors:** 


THE
### INSTITUTIONAL WORKER
**Being a Special Supplement to "The Hospital"**

### Editor's Notices.

**Contributions:**

Contributions should be written, or preferably typed, on one side of the paper only, and all articles sent in are accepted upon the distinct understanding that they are forwarded to The Hospital only.

The Editor cannot undertake to return MSS. not used, but when a stamped directed envelope is enclosed the MSS. may be returned if a special request is made.

Accepted articles and paragraphs of news will be paid for after publication at the scale rate.

**Address:**

To prevent delay all contributions and letters on editorial business must be addressed exclusively to the Editor, "The Hospital," 29 Southampton Street, Strand, London, W.C. It is important that this regulation shall be strictly observed.

**Correspondence:**
Correspondence on all subjects is invited. The name and address of correspondents must be given as a guarantee of good faith, but not necessarily for publication.

**Special Articles:**
Special articles are invited, and questions, inquiries, and paragraphs upon all matters relating to the work, administration, and management of general, special, mental, fever, cottage and convalescent hospitals, sanatoria, homes, institutions, societies and organisations for the treatment or care of the sick, injured and dependants of all classes. Every matter affecting the interests and welfare of the staff of all grades working in these institutions will receive special consideration.

**Administrative Medicine, Research and Items of News:**

Special terms will be paid for approved articles on subjects in this wide field by experts who have made some section of it their special study and interest. We wish to give prominence to every movement tending to advance accuracy of diagnosis, efficiency in treatment, and the development of modern methods to eradicate disease and promote the welfare of the suffering.

**A Bureau of Information:**

We invite inquiries and applications for information and help in providing for that numerous class of sufferers who require special care or house-room which they cannot obtain in their own homes or secure for themselves. **We cannot, however, prescribe, or recommend practitioners.**

**Books for Review:**

Publishers are particularly requested to send advance proofs of any new books of importance whenever possible, as the Editor has made arrangements to publish immediate reviews on a new plan.

**Photographs, Plans, Blocks, and Illustrations:**

It is requested that wherever possible MSS. may be accompanied by illustrations in any of the above forms. The name of the sender and of the article to which it belongs should be written on the back of each photograph, drawing, or block for purposes of identification.

**Local Papers:**

Newspapers containing reports or news paragraphs **should be marked** and addressed to the Sub-Editor.

**The Coupon System:**

A coupon will be found at the bottom of the third inside page of the cover each week. This coupon must be attached to every question or inquiry to which an answer is desired in The Hospital.

### A Help to Employment.

The greatly increasing number of announcements that appear from week to week in "The Institutional Worker" is undeniable evidence of the exceptional value of this section of The Hospital as a medium for all classes of Institutional Notices. Its usefulness requires no more adequate proof than the fact that the most important Institutions throughout the country always employ "The Institutional Worker" whenever they have a Vacancy to fill upon their Staffs. It will be obvious, therefore, that this Supplement affords unique facilities to those seeking employment in any grade or department of the Institution, Health and Sanitary Services, in consequence of its extensive distribution among Institutions of every description throughout the United Kingdom. For reaching the responsible Officials of these establishments "The Institutional Worker" provides a means at once appropriate and effective, since no other Journal possesses so comprehensive a circulation in these directions as The Hospital.

If you cannot find an appointment in this week's issue, suited to your capabilities, an announcement of twenty-one words, costing the nominal sum of 1s., in the Appointments Wanted column, will enable you to bring your requirements prominently to the notice of those likely to need your services.

The form printed below is for the convenience of our readers who wish to avail themselves of the incomparable utility of "The Institutional Worker," and when filled up should be posted with remittance to

*The Manager*, The Hospital,
28 and 29 Southampton Street,
Strand, W.C.

Appointments Wanted, 21 words ... 1s. 0d.

| | | |
|---|---|---|
| | | |
| | | |
| | | |
| | | |
| | | |
| | | |
| | | |

### Manager's Notices.

**All letters on business matters must be addressed to The Manager, "The Hospital," 28 and 29 Southampton Street, London, W.C.**

**Subscriptions,** which may commence at any time, are payable in advance. Cheques and Post-Office Orders should be crossed London County and Westminster Bank, Covent Garden Branch, and made payable to the Manager of The Hospital.

Rates of Subscription (including Postage).

| *United Kingdom.* | | *Foreign and Colonial.* | |
|---|---|---|---|
| Three Months ... ... | 2s. 0d. | Three Months ... ... | 2s. 6d. |
| Six Months ... ... | 4s. 0d. | Six Months ... ... | 5s. 0d. |
| Twelve Months ... ... | 6s. 6d. | Twelve Months ... ... | 8s. 8d. |

**Advertisements:**
To ensure insertion, all advertisements for The Hospital must reach the Manager not later than Wednesday morning in each week. For scale of charges see pages v. and 2.

### OFFICIAL APPOINTMENTS VACANT.

### Poor-Law Vacancies.

CHARGE NURSE (£30), Loughborough Union. A. W. Jarratt, C. to G., Union Offices, Loughborough. June 25.
CHARGE NURSE (£28), Tonbridge Union. N. R. Stone, C. to G., 23 Church Road, Tunbridge Wells. June 27.
NIGHT SUPERINTENDENT AND MASSEUSE (£38), Brentford Union. W. Stephens, C. to G., Union Offices, Isleworth, W. July 2.
PROBATIONER NURSE, Derby Union. N. Twigge, C. to G., Poor Law Offices, Derby. June 27.
RELIEVING OFFICER, ETC. (£70, commission and fees, about £82), Abergavenny Union. Mr. W. H. P. Scanlon, C. to G., Abergavenny. June 13.
STAFF NURSE (£25), Poplar and Stepney Sick Asylum. Mr. W. R. Foskett, Clerk to the Managers, Devons Road, Bromley-by-Bow, E. June 4.
STAFF NURSE (£22), Camberwell Guardians. Mr. C. S. Stevens, C. to G., Peckham Road, S.E. June 13.
STAFF NURSE (£30), Derby Union. N. Twigge, C. to G., Poor Law Offices, Derby. June 14.
STOCKTAKER (£2 2s. per visit), West London School District. Mr. F. G. Beeching, Clerk to the Managers, Ashford, Middlesex. June 3.
SUPERINTENDENT NURSE (£50), Dudley Union. Mr. E. Allen, C. to G., Dudley. June 5.
SUPERINTENDENT NURSE (£50), Pontypridd Union. W. Spickett, C. to G., Union Offices, Pontypridd. June 6.
SUPERINTENDENT OF LAUNDRY (£35), Norwich Guardians. Mr. E. J. W. Huggins, C. to G., Norwich. June 4.
TEMPORARY CASE-PAPER CLERK (£90), Wakefield Union. Mr. H. Beaumont, C. to G., Wakefield. June 4.

### Institutional and Administrative Vacancies.

MATRON (£40), Carnarvon Cottage Hospital. W. S. Jones, Hon. Secretary, 25 Market Street, Carnarvon. June 12.
MATRON (40), Hull Hospital for Women and Orthopædics. W. J. Stuart, Hon. Sec., Imperial Chambers, Bowlalley Lane, Hull. June 1.
MATRON (£120), Leicester Infirmary and Children's Hospital. H. Johnson, Secretary. June 10.
THEATRE SISTER (£30), King Edward VII. Hospital, Cardiff. E. A. Mont Wilson, Matron. June 6.

### Municipal Vacancies.

ASSISTANT SANITARY INSPECTOR (£120), Heston and Isleworth U.D.C. Mr. H. J. Baker, Clerk to the Council, Hounslow. June 14.
COST CLERK (Electricity Department) (£120), Hampstead B.C. Mr. A. P. Johnson, Town Clerk, Hampstead, N.W. June 3.
ELECTRICITY MAINS ENGINEER (£220), Hampstead B.C. Mr. A. P. Johnson, Town Clerk, Hampstead, N.W. June 3.
FOREMAN FOR HOUSE REFUSE REMOVAL (£24s. weekly), Bilston U.D.C. Mr. V. Turner, Engineer, Town Hall, Bilston. June 3.
HEALTH VISITOR (£75), Smethwick T.C. Mr. W. Shakespeare, Town Clerk, Smethwick. June 3.
INSPECTOR OF NUISANCES (£120 and fees), Isle of Thanet R.D.C. Mr. C. Taylor, Clerk to the Council, Minster, near Ramsgate. June 6.
INSPECTOR OF NUISANCES, ETC. (£70), Diss U.D.C. Mr. H. O. Lyus, Clerk to the Council, Diss. June 8.
INSPECTOR OF NUISANCES (£120 and fees), Isle of Thanet R.D.C. Mr. C. Taylor, Clerk to the Council, Minster, near Ramsgate. June 6.
JUNIOR ASSISTANT CLERK (£90), Wealdstone U.D.C. Mr. R. J. Bryant, Clerk to the Council, Wealdstone, Middlesex. June 4.
JUNIOR ASSISTANT FOR PUBLIC LIBRARY (£40), St. Pancras B.C. Mr. C. H. F. Barrett, Town Clerk, Town Hall, Pancras Road, N.W. May 29.
LADY INSPECTOR (£80), Warrington T.C. Dr. J. C. Hibbert, Medical Officer of Health, Warrington. June 8.
MATRON FOR FEVER HOSPITAL (£60), Wirral Joint Hospital Board. Mr. J. E. S. Ollive, Clerk to the Board, 54 Hamilton Street, Birkenhead. June 5.
NURSE MATRON (£40), Bridlington Borough Sanatorium. A. P. Matthewman, Town Clerk, Town Hall, Bridlington. May 28.
SURVEYOR (£200), Carmarthenshire C.C. Mr. J. W. Nicholas, Clerk to the Council, Carmarthen. May 29.
SANITARY INSPECTOR (£120), Chelsea B.C. Mr. T. Holland, Town Clerk, King's Road, Chelsea, S.W. June 13.
SURVEYOR AND INSPECTOR OF NUISANCES (£95), Ottery St. Mary U.D.C. Mr. H. Hartley, Clerk to the Council, Ottery St. Mary. June 3.
SURVEYOR'S GENERAL ASSISTANT (£100), Malden and Coombe U.D.C. Mr. J. W. Johnson, Clerk to the Council, New Malden. May 31.
TOWN CLERK (£750), Rotherham T.C. Mr. W. J. Board, Town Clerk, Rotherham. May 31.

### Miscellaneous Vacancies.

SCHOOL ATTENDANCE OFFICERS (£80), L.C.C. Education Offices, Victoria Embankment, W.C. June 1.
SCHOOL NURSE (£80), Dewsbury Education Committee. S. G. Bibby, Secretary, Education Office, Dewsbury. May 31.
EDUCATION INSPECTOR (£150), Norfolk Education Committee. Mr. T. A. Cox, Secretary for Education, Shire Hall, Norwich. June 6.

